# Human exposure to Crimean-Congo hemorrhagic fever virus is associated with livestock management and handling practices in southcentral Tanzania, 2023–2024

**DOI:** 10.1186/s12879-026-13919-x

**Published:** 2026-07-09

**Authors:** Cheryl Meehan, Grace Mwangoka, Mwokozi Mwanzalilla, Woutrina A. Smith, Maxmillian Mpina, Macdonald Farnham, Robinson Mdegela, Robert Sumaye, Silas Temu, Ali Mohamed Ali, Hajirani Msuya, Jumaa Athumani Jumaa, Huruma Sayale, Jane Orotta, George Makingi, Sijali Zinkankuba, Ahmed Amasha, Amina Abdallah Kinyogori, Yumba Yekonia, Erin Howell, Phoebe Lawton, Tracy Drazenovich, Alexandre Tremeau-Bravard, Sonja Montanus, Brian H. Bird

**Affiliations:** 1 One Health Institute, Weill School of Veterinary Medicine, University of California, Davis, 1089 Veterinary Medicine Drive, Davis, CA 95616 USA; 2 Ifakara Health Institute, P.O. Box 78 373, Dar es Salaam, Tanzania; 3 Department of Veterinary Medicine and Public Health, Sokoine University of Agriculture, P.O. Box 30 007, Morogoro, Tanzania

**Keywords:** Tickborne disease, Crimean-Congo hemorrhagic fever, Zoonotic disease, Tanzania

## Abstract

**Background:**

Crimean-Congo hemorrhagic fever virus (CCHFV) is a tick-borne zoonotic pathogen widely distributed across Africa, Asia, and Europe. Human infection occurs through tick bites or contact with infected animal tissues. In Tanzania, evidence of human exposure exists, but data on population-level seroprevalence and associated behavioral risk factors remain limited. This study assessed human CCHFV seroprevalence and identified demographic, behavioral, and environmental factors associated with prior exposure in livestock-keeping communities in southcentral Tanzania.

**Methods:**

A community-based cross-sectional study was conducted in four districts of southcentral Tanzania during 2023–2024. A total of 850 adults completed structured questionnaires on demographic and behavioral factors and provided blood samples. Serum samples were tested for CCHFV IgG antibodies using a commercial ELISA. Logistic regression models were used to identify factors associated with seropositivity.

**Results:**

Overall CCHFV seroprevalence was 3.41% (29/850). Seroprevalence increased with age and was highest among participants aged ≥ 51 years (6.28%, *p* = 0.044). In multivariable analysis, three factors were significantly associated with seropositivity: households with three or more children under five years of age (adjusted OR = 3.45, 95% CI 1.13–10.53, *p* = 0.030); butchering and consuming animals that died of illness (adjusted OR = 5.48, 95% CI 1.96–15.36, *p* = 0.001); and grazing animals on private land without other livestock, which was protective (adjusted OR = 0.32, 95% CI 0.12–0.90, *p* = 0.030).

**Conclusions:**

Human CCHFV exposure in southcentral Tanzania is associated with household composition, animal-handling practices, and grazing behaviors. These findings highlight modifiable behavioral risk factors that may inform targeted public health interventions. As this study focuses on human serologic evidence of prior exposure, further integrated human–animal investigations are needed to better understand transmission dynamics.

**Supplementary Information:**

The online version contains supplementary material available at https://doi.org/10.1186/s12879-026-13919-x.

## Background

Crimean-Congo hemorrhagic fever (CCHF) is a tick-borne viral zoonosis that causes severe disease in humans, with potentially fatal consequences, across Africa, the Middle East, Asia, and parts of Europe. Crimean-Congo hemorrhagic fever virus (CCHFV) is a member of the genus *Orthonairovirus* (family *Nairoviridae*) and was first described in Crimea in the 1940s [1]. The virus is maintained in nature through a tick–vertebrate cycle, with ticks acting as both vector and reservoir through transstadial and transovarial transmission [2, 3]. Although transmission typically occurs through the bite *Hyalomma* ticks, CCHFV is carried by greater than 35 tick species [3–5]. A wide range of domestic and wild vertebrates including cattle, sheep, and goats develop transient viremia without clinical disease, facilitating continued viral circulation [2, 6].

In humans, CCHFV infections are acquired primarily through the bites of infected ticks or contact with the blood, organs, or bodily fluids of infected livestock [7–9]. These exposures commonly arise during slaughtering, butchering, veterinary care, and animal husbandry activities, placing farmers, herders, abattoir workers, and veterinarians at increased risk of exposure [10]. Human-to-human transmission of CCHFV has been documented, particularly in healthcare settings, through contact with infected blood or bodily fluids [11]. Clinical presentation ranges from mild, nonspecific febrile illness to severe hemorrhagic fever. While the majority of human cases are subclinical, approximately one in eight clinical cases progress to severe hemorrhagic fever, with case fatality rates of 10–40% in hospitalized patients [12–14].

Globally, CCHF is reported across more than 30 countries in Africa, Asia, and Europe, with an estimated 10,000–15,000 human infections and about 500 deaths annually [15]. Seroprevalence studies suggest that human exposure rates may be as high as 22.5% [16]. In Africa, 494 cases and 115 deaths occurred during outbreaks between 1956 and 2020, and since 2020, nine African countries have reported their first cases [17]. The rising incidence of CCHF is attributed to intensified livestock trade and movements, climate change, expanding tick ranges, and reduced surveillance and vector control during the COVID-19 pandemic, all of which have heightened human exposure risks to infected ticks and animals [15, 16].

In Tanzania, available evidence indicates that CCHFV circulation is present but underrecognized. Across-sectional human–livestock survey in northern Tanzania identified human seroprevalence of 15.1%, alongside livestock seropositivity of 49.6% in cattle, 33.8% in goats, and 27.8% in sheep [18]. However, no confirmed human outbreaks have been reported in Tanzania, in contrast to neighboring Uganda where multiple outbreaks have been confirmed since 2013 [18]. A nationwide human serosurvey (2017–2018) in Tanzania found approximately 1% human seropositivity, with marked geographic variation across the country [19]. Additionally, a hospital-based study of febrile patients in northern Tanzania (2012–2014) found 1.8% seropositivity and linked risk in people to livestock-related exposures [20].

Together, these findings indicate that human exposure to CCHFV occurs in Tanzania, particularly in pastoral and agropastoral settings, but may be undetected due to limited surveillance. To address this gap, we conducted a livestock-keeping community-based study in southcentral Tanzania, to estimate human CCHFV seroprevalence and identify demographic, behavioral, and environmental factors associated with prior exposure. Understanding these patterns can inform targeted surveillance and behavior-focused intervention strategies.

## Methods

A community-based, cross-sectional study was carried out from November 2023 to April 2024, in four districts of southcentral Tanzania. These included Iringa Rural and Kilolo Districts in the Iringa Region, as well as the Kilombero and Mlimba Districts in the Morogoro Region. These regions are known for their robust agricultural sector, with farming and livestock rearing as the primary economic activities. The selected regions represent floodplain and subtropical highland ecosystems, and include open space, grazing areas, and national parks where wildlife, livestock, and humans co-mingle.

### Ethics approvals and consent to participate

The study was conducted in accordance with the Declaration of Helsinki the International Conference on Harmonization Good Clinical Practices (ICH GCP) and the principles of the Belmont Report. Prior to initiation of this study, ethical and regulatory review and approval was obtained from the appropriate authorities in both Tanzania and the United States for the proposed study protocols including the Institutional Review Boards at the Ifakara Health Institute (IHI/IRB/No:27-2023 and IHI/IRB/EXT/No: 17-2025) and the University of California, Davis (UCD IRB 2086677-1), and the National Institute of Medical Research of Tanzania (NIMR/HQ/R.8a/Vol.1X/4324 and NIMR/HQ/R.8c/Vol.I/2527). Participation was completely voluntary and written informed consent was obtained prior to participant enrollment and data collection. All participant data was de-identified to protect participant privacy prior to analyses and individuals were potentially eligible to participate if aged ≥ 18 years.

### Recruitment and enrollment

The study team collaborated with village leaders to organize community sensitization meetings, where the study objectives, enrollment procedures, and eligibility criteria were explained. The project team, together with community and pastoralist leaders, identified potential participants from agro-pastoralist and pastoralist populations, as well as butchers and veterinarians / livestock officers, who are at risk of acquiring the infection. During these meetings, potential participants were assured of the voluntary nature of participation and the confidentiality of their information. Cross-sectional community enrollment was utilized across all study sites. Study sites included the Iringa Region: Iringa Rural District (4 wards = Idodi, Mahuninga, Mlenge, Mlowa) and Kilolo District (1 ward = Udekwa) and the Morogoro Region: Kilombero District (1 ward = Signal) and Mlimba District (2 wards = Ching’anda, Chita).

### Social demographic data and biological sample collection

After obtaining consent, participants completed a structured questionnaire and provided blood samples. The questionnaire was based on previous zoonotic disease surveillance tools and pretested for clarity and interviewer understanding. It captured socio-demographic characteristics and occupational, personal protective, and dietary behaviors that could facilitate transmission and spread of CCHFV (see: Supplementary File 1). Data were collected electronically using KoboToolbox on Android tablet devices (Samsung Galaxy).

A maximum of 6 mL of whole venous blood samples for serum separation were collected by trained phlebotomists, nurses, or physicians using standard venipuncture from the right or left antebrachium. Samples were centrifuged to separate serum, which was then aliquoted into two vials to reduce freeze–thaw cycles during testing. Serum samples were stored in liquid nitrogen in the field and subsequently transferred to the Ifakara Health Institute (IHI) laboratory in Bagamoyo, where they were maintained at − 80 °C until serological analysis. Prior to testing, all specimens were heat inactivated at 56 °C for 30 min. Serological testing for CCHFV exposure (as determined by IgG antibodies) was performed using the ID Screen^®^ CCHF Double Antigen Multi-Species ELISA Kit (Innovative Diagnostics, France; https://www.innovative-diagnostics.com) following the manufacturers recommended protocol. Results were expressed as S/P% (S/P% = (optical density of sample divided by optical density of positive control) x 100). Samples were considered positive if S/P% was greater than 30%.

### Data analysis

The primary outcome was anti-CCHFV specific IgG antibody seropositivity (defined as 0 for negative and 1 for positive). Seropositivity reflects prior exposure to CCHFV and does not indicate timing, source, or transmission pathway of infection. Independent variables included both demographic and behavioral factors retrieved from the survey data set (Tables 1 and 2). Age was converted to categories (18–31; 32–41; 42–51; 51+). Occupation status was recoded as either “High” or “Low” risk. High risk occupations included: meat processing, livestock keeping, hunting, and crop farming. All other occupations were considered low risk. Behavioral risk factors included dietary practices, and animal care/slaughter practices. Where categorical behavioral risk factors included more than two choices, responses were recoded as binary with the hypothesized highest risk option being compared to all other options as a group.

**Table 1:** Demographic characteristics of the study participants

Variables	Level	*n* (%)
Numbers of study participants	Overall	850
Sex	Male	650 (76.5)
	Female	200 (23.5)
Age category (years)	18–31	220 (25.9)
	32–41	214 (25.2)
	42–50	209 (24.6)
	51+	207 (24.4)
Household size	1–5	464 (54.6)
	6–10	282 (33.2)
	> 10	104 (12.2)
Number of children under 5 in household	0	318 (37.4)
	1–2	443 (52.1)
	> 2	88 (10.4)
Occupation*	High-risk	347 (40.8)
	Low-risk	497 (58.5)

* High risk occupations included meat processing, livestock keeping, hunting, and crop farming. All other occupations were considered low risk

**Table 2:** Behavioral determinants and potential risks of exposure to zoonotic pathogens

Variables	Level	*n* (%)
Consume raw meat	No	776 (91.3)
	Yes	74 (8.7)
Consume raw milk	No	543 (63.9)
	Yes	207 (36.1)
Bushmeat consumption in the past 12 months	No	45 (22.7)
	Yes	153 (77.3)
Animal ownership	No	261 (30.7)
	Yes	589 (69.3)
Number of species of animals owned	1–3	372 (63.2)
	> 3	217 (36.8)
Location where animal slaughtered/butchered*	Formal	103 (20.7)
	Informal	395 (79.3)
Use of viscera	Take home and consume	233 (39.6)
	Other use	312 (53.0)
Place where animals are grazed	Public land	83 (14.1)
	Reserve	31 (5.3)
	Village/Community	248 (42.1)
	Private land without other animals	208 (35.3)

*Formal locations include dedicated abattoirs and slaughterhouse facilities

Prevalence of CCHFV was calculated in the context of several demographic factors including ward, sex, age, household data, and occupation. To determine the association between seropositive status and demographic or behavioral risk factors at the univariable level, a chi-square test or Fisher exact test was used. Variables with an association of *p* < 0.25 were retained for further analysis. Multivariable logistic regression models were built using forward selection. Model fit was tested using Akaike Information Criterion (AIC) and Bayesian Information Criterion (BIC) indicators. ROC AUC was used in final model selection. Odds ratios (ORs) and 95% confidence intervals (CIs) were obtained by exponentiating the model coefficients and confidence bounds. Statistical analyses were conducted using STATA (version 17; Stata Corp, College Station, TX, USA), and R Statistical Software (version 3.4.3, https://www.r-project.org/) was used for graphical analysis.

Geographic data analyses were conducted using R Statistical Software (version 3.4.3, https://www.r-project.org/) and data visualization was conducted in R using the sf [21, 22], ggplot2 [23], ggspatial [24], and cowplot packages [25]. Ward-level seroprevalence of CCHFV was calculated from individual test results and aggregated by administrative unit. Seroprevalence was defined as the proportion of seropositive individuals out of the total tested within each ward. Administrative boundary shapefiles for wards were sourced from the 2022 Tanzanian census dataset and read using the sf package. Ward names were harmonized to match survey data, and boundaries were merged with prevalence estimates.

## Results

Table 1 reports demographic characteristics of the study population. A total of 850 participants were enrolled from communities representing mixed livelihood systems. The sex distribution was skewed toward males (76.5% male, 23.5% female). Although the study population was predominantly male, CCHFV seroprevalence was similar among male (3.5%) and female (3.0%) participants. Therefore, sex imbalance is unlikely to explain the primary associations observed. Participants were evenly distributed across age categories. Households with children under five years were common (62.5%). High-risk occupations, including meat processing, livestock keeping, hunting, and crop farming, were reported by 40.8% of participants.

**Fig. 1:**
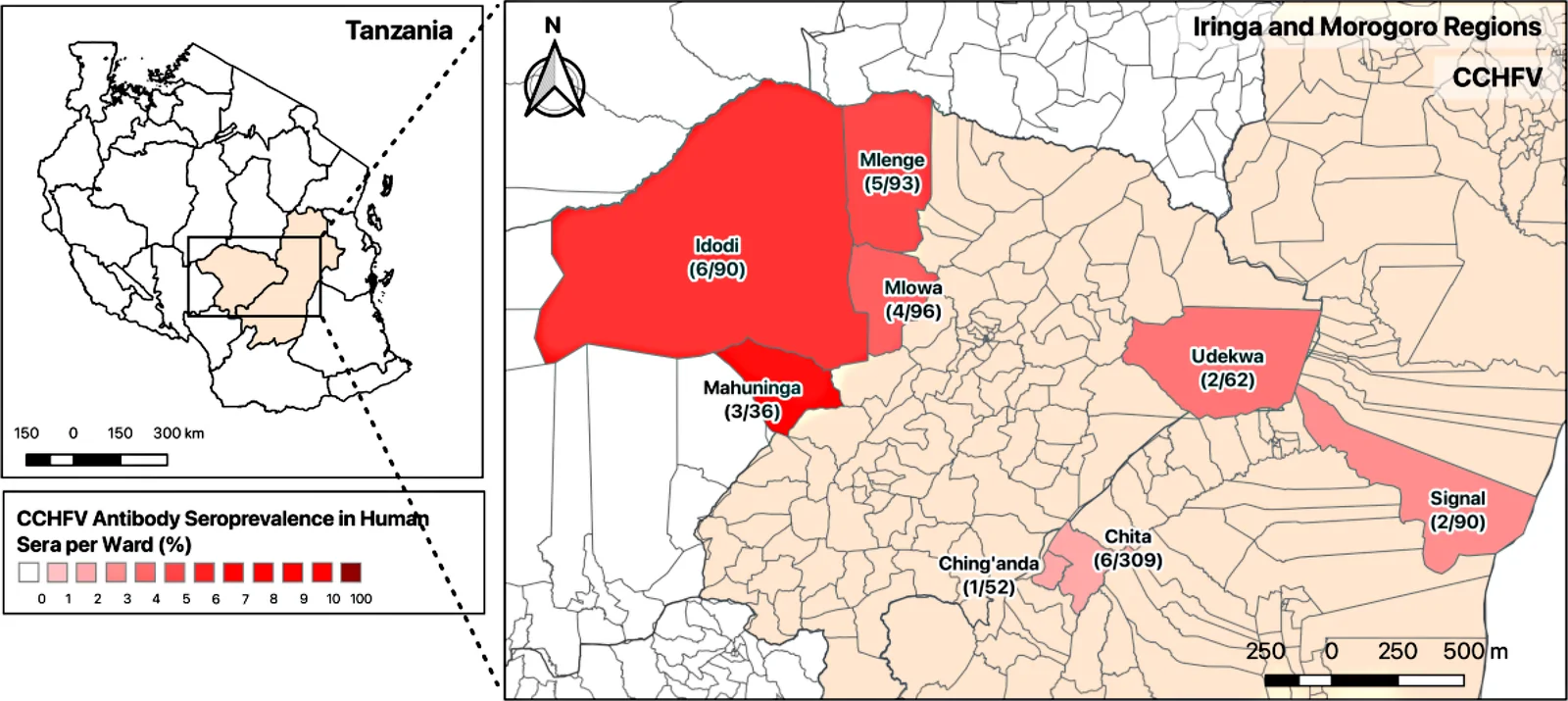
Geographic distribution by ward of Crimean-Congo hemorrhagic fever virus (CCHFV) IgG seroprevalence among human community study participants in central Tanzania, 2023–2024. Left: Tanzania map displaying ward boundaries with sampled wards shown in tan. Right: Choropleth maps showing CCHFV IgG seroprevalence in red scale by ward

Figure 1 shows the geographic distribution by district and ward of Crimean-Congo hemorrhagic fever virus (CCHFV) IgG seroprevalence. Mean prevalence was highest in the Iringa Rural District (mean seroprevalence = 5.36; range = 4.17–8.33) and lowest in the Mlimba District (mean seroprevalence = 1.93; range = 1.92–1.94). Kilolo (seroprevalence = 3.22) and Kilombero Districts (seroprevalence = 2.22) were represented by one ward each.

Table 2 presents behavioral determinants associated with potential zoonotic exposure. Most respondents reported not consuming raw meat (91.3%), while 8.7% did. Consumption of raw milk was reported by 36.1%, and bushmeat consumption within the past year by 77.3%. Informal slaughter settings were predominant (79.3%), and 39.6% reported taking viscera home for consumption.

Table 3 summarizes risk-reduction practices related to zoonotic diseases such as CCHF. Only 9.2% practiced quarantine for newly acquired animals. Most reported no use of personal protective equipment (PPE, 73.5%). When animals became sick, 6.1% of people reported slaughtering the animals for consumption; however, when animals died, 46.9% reported butchering and consuming animals.

**Table 3:** Community member participation in potential risk reduction strategies for zoonotic pathogens

Variables	Level	*n* (%)
Is there a quarantine period for new animals?	No	535 (90.8)
	Yes	54 (9.2)
Use of personnel protective equipment (PPE)	No PPE	433 (73.5)
	One form of PPE	134 (22.8)
	More than one form of PPE	22 (3.7)
How often are animals treated for ticks?	As needed	210 (47.3)
	Daily	3 (0.7)
	Weekly	141 (31.8)
	Monthly	88 (19.8)
	Missing	2 (0.5)
What do you do when an animal gets sick?	Other*	553 (93.9)
	Slaughter and consume the meat	36 (6.1)
What do you do when an animal dies of illness?	Other‡	313 (53.1)
	Butcher and consume the meat	276 (46.9)

*kill animal and dispose the carcass, sell the live animal, get veterinary care, report to authorities, do nothing; ‡bury/dump the carcass, feed the carcass to dogs

Table 4 summarizes CCHFV seroprevalence across demographic and geographic groups of people. The overall seroprevalence was 3.41% (29/850). Seroprevalence increased with age and was highest in Iringa Rural District (5.36%). Table 5 presents associations between CCHFV seroprevalence and selected demographic and behavioral characteristics. Household size, number of children under five, occupation type, animal ownership, grazing location, and carcass-handling practices were significantly associated with infection risk.

**Table 4:** CCHFV human seroprevalence by demographic and geographic factors

**Age (** ***p*** ** = 0.044)**	**Positive**	**Negative**	**Total**	**Prevalence**
18–31	6	214	220	2.73
32–41	3	211	214	1.40
42–50	7	202	209	3.35
51+	13	194	207	6.28
**Sex**	**Positive**	**Negative**	**Total**	**Prevalence**
Male	23	627	650	3.50
Female	6	194	200	3.00
**District (** ***p*** **= 0.089)** Ward	**Positive**	**Negative**	**Total**	**Prevalence**
**Iringa Rural**	**18**	**218**	**336**	**5.36**
Idodi	6	84	90	6.67
Mahuninga	3	33	36	8.33
Mlenge	5	88	93	5.38
Mlowa	4	92	96	4.17
**Kilolo**	**2**	**60**	**62**	**3.22**
Udekwa	2	60	62	3.22
**Kilombero**	**2**	**88**	**90**	**2.22**
Signal	2	88	90	2.22
**Mlimba**	**7**	**355**	**362**	**1.93**
Ching’anda	1	51	52	1.92
Chita	6	303	309	1.94
**Grand Total**	**29**	**821**	**850**	**3.41**

**Table 5:** Human seroprevalence of CCHFV by individual demographic and behavioral characteristics

			CCHF seroprevalence				Total		*p*-value
			Positive (%)	Negative (%)					
Household size									
1–4			6 (1.8)	334 (98.2)		340			0.018
5–7			10 (3.3)	294 (96.7)		304			
8+			13 (6.3)	193 (93.7)		206			
Number of children under age 5 in the household									
0			7 (2.2)	311 (97.8)		318			< 0.001
1–2			12 (2.7)	431 (97.3)		443			
3+			10 (11.4)	78 (88.6)		88			
Occupation^									
Low-risk group			12 (2.4)	485 (97.6)		497			0.051
High-risk group			17 (4.9)	330 (95.1)		347			
Consume raw meat									
No			24 (3.1)	752 (96.9)		776			0.099
Yes			5 (6.8)	69 (93.2)		74			
Bushmeat consumption in the past 12 months									
No			2 (4.4)	43 (95.6)		45			0.319
Yes			3 (2.0)	150 (98.0)		153			
Do you own any animals?									
No			2 (0.8)	259 (99.2)		261			0.005
Yes			27 (4.6)	562 (95.4)		589			
Number of animals owned									
1–3			10 (2.7)	362 (97.3)		372			0.004
4+			17 (7.8)	200 (92.2)		217			
Animals grazed on private land without other livestock/animals									
No			22 (5.8)	359 (94.2)		381			0.062
Yes			5 (2.4)	203 (97.6)		208			
What do you do when an animal gets sick?									
Other*			24 (4.3)	529 (95.7)		553			0.224
Slaughter and consume			3 (8.3)	33 (91.7)		36			
What do you do when an animal dies of illness?									
Other‡			5 (1.6)	308 (98.4)		313			< 0.001
Butcher and consume			22 (8.0)	254 (92.0)		276			

^ High risk occupations included meat processing, livestock keeping, hunting, and crop farming. All other occupations were considered low risk

*kill animal and dispose the carcass, sell the live animal, get veterinary care, report to authorities, do nothing ‡bury/dump the carcass, feed the carcass to dogs

In the final multivariable model (Table 6), three factors remained significantly associated with infection: households with three or more children under five (adjusted OR = 3.45, 95% CI 1.13–10.53, *p* = 0.030); butchering and consuming animals that died of illness (adjusted OR = 5.48, 95% CI 1.96–15.36, *p* = 0.001); and grazing animals on private land without other livestock, which was protective (adjusted OR = 0.32, 95% CI 0.12–0.90, *p* = 0.030). Age was included in the final model based on AIC, BIC, and ROC AUC outcomes. The model including age has a lower AIC (191.9654) than the model excluding age (193.5874). Conversely, the model excluding age has a lower BIC (215.3505) than the model including age (226.7864). The model including age demonstrates a higher ROC AUC (0.81) than the model excluding age (0.77). While the BIC prefers the model excluding age for parsimony, we chose the model including age for the improvement overall fit considering complexity (AIC) and the improvement in predictive performance (ROC AUC).

**Table 6:** Multivariable logistic regression results for factors associated with CCHFV IgG seropositivity among human participants in southcentral Tanzania

Variable		Univariable analysis		Multivariable analysis	
	*N*	Odds ratio (95% CI)	*p*-value	Odds ratio (95% CI)	*p*-value
**Age category (years)**	850				
18–31		1		1	
32–41		0.51 (0.13–2.05)	0.341	0.54 (0.12–2.38)	0.413
42–50		1.24 (0.41–3.74)	0.708	1.36 (0.39–4.74)	0.634
51+		2.39 (0.89–6.41)	0.083	2.59 (0.85–7.92)	0.095
**Number of children in the household**	849				
No. under 5		1		1	
1–2		1.24 (0.48–3.18)	0.659	1.03 (0.36–2.98)	0.953
3+		5.70 (2.1–15.44)	0.001	3.45 (1.13–10.53)	**0.030**
**Graze animal in private land without other livestock/animals**	589				
No		1		1	
Yes		0.40 (0.15–1.08)	0.07	0.32 (0.12–0.90)	**0.030**
**What do you do when animal die?**	589				
Other‡		1		1	
Butcher and consume the meat		5.34 (1.99–14.29)	0.001	5.48 (1.96–15.36)	**0.001**

*kill animal and dispose the carcass, sell the live animal, get veterinary care, report to authorities, doing nothing

‡bury/dump the carcass, feed the carcass to dogs

## Discussion

This study provides one of the few community-based assessments of Crimean-Congo hemorrhagic fever virus (CCHFV) exposure among livestock-keeping populations in Tanzania, highlighting important demographic, behavioral, and environmental factors that shape infection risk. The overall seroprevalence of 3.4% is over 3 times higher than the most recent national level serosurvey (20), suggesting that levels of human exposure may be higher in rural settings. Seropositivity was highest among older adults and individuals residing in Iringa Rural District, pointing to cumulative exposure and localized ecological factors that may support virus maintenance. Behavioral and demographic patterns, particularly household composition, carcass-handling practices, and shared grazing systems, were strongly associated with infection risk, underscoring the role of human-animal interface behaviors as correlates of exposure risk in this population. The presence of more young children could reflect higher household tick exposure, or greater caregiver burden in high-risk environments, which could increase the adult’s risk of CCHFV infection. Conversely, protective effects were observed where animals grazed on private land, indicating that limiting herd mixing may reduce tick-borne transmission. Together, these findings emphasize that both household-level practices and broader environmental management influence disease exposure dynamics, reinforcing the need for integrated One Health approaches to mitigate CCHFV risk in endemic regions.

Practices that increase opportunities for contact with infected animals or ticks, as well as exposure to blood and tissues during animal handling and slaughter, are known to increase risk for CCHFV exposure. The significant association between butchering and consuming animals that died of illness underscores the potential for direct transmission through unsafe carcass-handling practices. Similar risk behaviors related to slaughter and meat processing have been documented in Iran, where livestock-related occupational exposures contributed to human seroprevalence [26]. Conversely, the low uptake of personal protective equipment (PPE) use, and the absence of quarantine procedures for newly introduced animals, highlight critical gaps in preventive practices that may facilitate ongoing viral circulation within herds and households.

Environmental and land-use factors appear to play a pivotal role in CCHFV transmission.

Participants whose animals grazed on private land without other livestock had significantly lower odds of infection, suggesting that reduced inter-herd contact may limit tick exchange and viral amplification. By contrast, the use of shared grazing areas in this study reflects patterns previously observed in Afghanistan, where communal pastures contribute to sustained tick populations [27]. The geographic clustering of higher seroprevalence in Iringa Rural District may reflect localized ecological factors such as vegetation type, livestock density, proximity to protected areas, and tick habitat suitability that may be associated with historical CCHFV exposure. These spatial patterns warrant further entomological and ecological investigation to clarify environmental drivers of transmission. However, because IgG seropositivity reflects prior exposure rather than active infection, these findings should not be interpreted as evidence of current viral circulation within the study area.

This study has several limitations that should be considered when interpreting the findings. First, behavioral data were self-reported and may be subject to recall bias or misclassification of exposures. Second, although multivariable modeling was used to account for measured demographic and behavioral factors, residual confounding from unmeasured variables remains possible. Variables such as wealth, occupation, access to livestock resources, herd size, land ownership, and other contextual factors may influence both exposure opportunities and participation in livestock-related activities. As a result, the reported associations should be interpreted as indicators of exposure patterns rather than evidence of direct causal relationships. Future studies incorporating a broader range of socioeconomic, ecological, and livestock management variables may help clarify the relative contribution of these factors to human infection risk.

Third, the study population was predominantly male; however, seroprevalence was similar between males and females and sex was not retained in the final model, suggesting that this imbalance is unlikely to explain the primary associations, though it may limit generalizability of behavioral findings. Finally, the use of IgG-based serological testing provides evidence of prior exposure but does not determine the timing or source of infection, nor does it confirm current viral circulation within the study area. Consequently, seropositivity should not be interpreted as evidence of current viral circulation or active transmission within the study area.

Confirmation of ongoing transmission would require contemporaneous detection of virus by or recent infection in humans, livestock, or tick vectors (e.g., RT-PCR or IgM ELISA assays). This study did not include concurrent sampling of livestock or tick vectors, limiting our ability to directly assess virus circulation in animal reservoirs or to identify transmission pathways. As such, observed associations should be interpreted as behavioral correlates of human exposure rather than causal mechanisms of transmission. Future studies integrating human, animal, and vector data integrating a more comprehensive sampling approach will be important for further understanding of CCHFV transmission dynamics in these settings.

Results from this work however do indicate the need for strengthening of public health based mitigation strategies that prioritize education and behavior change related to animal carcass disposal, reducing the consumption of sick or dead animals, increasing awareness regarding the use of protective clothing other biosafety measures, and encouraging the implementation of routine tick control among livestock. Strengthening veterinary extension services and promoting safe slaughter practices could substantially reduce exposure risk at the community level. Additionally, integrating CCHFV surveillance into broader One Health frameworks linking human, animal, and vector data would improve early detection and response capacities for both human and animal medicine. The observed associations between household characteristics and CCHFV seropositivity, community-level interventions should also address potentials for intra-household exposure, particularly among families with young children and premises with multiple animal species.

## Conclusion

Human CCHFV exposure in these agropastoral communities reflects a multifactorial risk landscape shaped by demographics, behavior, and the environment. Within the districts studied in southcentral Tanzania, the persistence of animal-handling practices without PPE, combined with shared grazing and limited protective measures from ticks and virus exposure, underscores the need for multisectoral approaches that link human health, veterinary services, and environmental management. Strengthening community awareness, improving access to veterinary services and public health resources, and reducing high-risk contact behaviors are critical steps toward mitigating CCHFV transmission in similar endemic settings.

## Supplementary Information

Below is the link to the electronic supplementary material.

Supplementary Material 1

## Data Availability

Datasets used and / or analyzed during the current study are available from the corresponding author upon reasonable request.

## Abbreviations

CCHF: Crimean-Congo Hemorrhagic Fever
CCHFV: Crimean-Congo Hemorrhagic Fever Virus
ELISA: Enzyme-Linked Immunosorbent Assay
IHC GCP: International Conference on Harmonization Good Clinical Practice
IHI: Ifakara Health Institute
ROC AUC: Receiver Operating Characteristic Area Under the Curve
AIC: Akaike Information Criterion
BIC: Bayesian Information Criterion
PPE: Personal Protective Equipment
