# Supplementary material for: Human exposure to Crimean-Congo hemorrhagic fever virus is associated with livestock management and handling practices in southcentral Tanzania, 2023–2024
Source: BMC Infect Dis. 2026 Jul 9;26:1767. doi: 10.1186/s12879-026-13919-x (PMC13640119; doi:10.1186/s12879-026-13919-x)
Supplement: Supplementary file 1 [file 12879_2026_13919_MOESM1_ESM.pdf]

Supplementary File 1: Survey instrument for the collection of demographic, behavioral, and CCHF risk factor data for human subjects in southcentral Tanzania.

| Question | Choices |
|---|---|
| ***Select Region*** | [1] Morogoro<br>[2] Iringa<br>[3] Pwani |
| ***Select District*** | [1] Bagamoyo<br>[2] Chalinze<br>[3] Iringa Rural<br>[4] Kilolo<br>[5] Kilombero<br>[6] Mlimba |
| ***Select Ward*** | [1] Fukayosi<br>[2] Kiwangwa<br>[3] Miono<br>[4] Lugoba<br>[5] Msata<br>[6] Idodi<br>[7] Mlowa<br>[8] Mlenge<br>[9] Mahuninga<br>[10] Udekwa<br>[11] Signal<br>[12] Chita<br>[13] Ching'anda |
| ***Select Village*** | [1] Masimbani<br>[2] Kikaro<br>[3] Mihuga<br>[4] Miono<br>[5] Kweikonje<br>[6] Mkwajuni<br>[7] Kipera<br>[8] Makao Makuu<br>[9] Mazizi<br>[10] Msata<br>[11] Pongwe Msungura<br>[12] Kihangaiko<br>[13] Mkoko<br>[14] Saleni<br>[15] Lugoba<br>[16] Lunga<br>[17] Mindutulieni<br>[18] Makombe<br>[19] Kinzagu<br>[20] Masuguru<br>[21] Mwetemo<br>[22] Bago<br>[23] Kiwangwa<br>[24] Msinune<br>[25] Mkenge<br>[26] Mwavi<br>[27] Fukayosi |

| Question | Choices | |
|---|---|---|
| | [28] Kidomole<br>[29] Idodi<br>[30] Mapogoro<br>[31] Tungamalenga<br>[32] kitisi<br>[33] Mafuluto<br>[34] Malizanga<br>[35] Kisanga<br>[36] Magombwe<br>[37] Kinyika<br>[38] Isele<br>[39] Mahuninga<br>[40] Makifu<br>[41] Kisilwa<br>[42] Udekwa<br>[43] Wotalisoli<br>[44] Ifuwa<br>[45] Sagamaganga<br>[46] Signal<br>[47] Chita<br>[48] Makutano<br>[49] Merera<br>[50] Msita<br>[51] Ching'anda<br>[52] Lufulu | |
| ***Consent form administered and signed?*** | [a] Yes<br>[b] No | |
| ***Human Survey*** | | Section Name |
| ***Location of interview?*** | [a] Home/residence<br>[b] Health clinic<br>[c] Community center<br>[d] Animal production site or abattoir<br>[e] Crop production site<br>[f] Temporary settlement<br>[g] Tourism site<br>[h] Protected area<br>[i] Village Gorverment Office<br>[j] Ward Executive Office<br>[k] Other | |
| ***Explain*** | [Text] | |
| ***Participant ID*** | [Integer] | |
| ***ID ya Mshiriki*** | [Calculation] | |
| ***Participant ID ${qn3}*** | | |
| ***Herd ID*** | [Integer] | |

| Question | Choices |
| --- | --- |
| ***Herd ID: ${Herd_ID}*** | |
| ***Date of interview*** | [Date] |
| ***Season of interview*** | [a] Rainy<br>[b] Dry |
| ***Participant's Gender*** | [a] Male<br>[b] Female |
| ***What is your age?*** | [Integer] |
| ***Body temperature*** | [Decimal] |
| ***Occupation [Multiple Choice]*** | [1] No source of income<br>[2] Casual labour(Kibarua)<br>[3] Donations<br>[4] Fishing<br>[5] Farming<br>[6] Livestock keeping<br>[7] Skilled labour(fundi)<br>[8] Driver<br>[9] Pension<br>[10] Salary<br>[11] Business<br>[12] Other |
| ***Explain*** | [Text] |
| . | Section Name |
| ***How many children younger than 5 years old live in the household?*** | [Integer] |
| ***How many household members aged between 5 to 18 years live in the household?*** | [Integer] |
| ***How many household members aged 18 years and above live in the household?*** | [Integer] |
| ***Total number of people live in your household is ${qn9}*** | |
| ***Have you travelled outside this district in the past 30 days?*** | [a] Yes<br>[b] No |
| ***What was the primary purpose of travel? [Multiple Choice]*** | [1] Work<br>[2] Visit friends or family<br>[3] Leisure/entertainment<br>[4] Other |
| ***Explain other reasons for travel*** | [Text] |

| Question | Choices |
| --- | --- |
| ***What was the start date of your most recent travel?*** | [Date] |
| ***Location of most recent travel? Short answer.*** | [Text] |
| ***What is the highest level of education that you have completed?*** | [a] No formal education<br>[b] Primary school<br>[c] Secondary school<br>[d] Technical school<br>[e] College/University/Professional school |
| ***Have you seen any ticks on an animal or person in the past 30 days?*** | [a] Yes<br>[b] No |
| ***Please check the box next to the image of any of the following types of ticks you have seen on an animal or person in the past 30 days. [Multiple Choice]*** | [1] Hyalomma<br>[2] Amblyomma<br>[3] Boophilus<br>[4] Rhipicephalus<br>[5] I am unsure of the specific type |
| ***In the last 30 days, how many times have you found a tick on your body, either attached or unattached?*** | [a] 0<br>[b] 1-5<br>[c] 6-10<br>[d] 11-12<br>[e] More than 20 |
| ***How do you remove ticks from pets, yourself, or other people? [Multiple Choice]*** | [1] Leave it / do not do anything with the tick<br>[2] Pull tick off with fingers<br>[3] Squeeze the tick and then pull to remove<br>[4] Pick tick off with fingers and then smash<br>[5] Other |
| ***Explain*** | [Text] |
| ***Have you ever eaten ticks?".*** | [a] Yes<br>[b] No |
| ***In the past 30 days, have you eaten any engorged, attached, or unattached ticks?*** | [a] Yes<br>[b] No |
| ***Are you aware of any of the youth in your family or neighbors' families eating ticks?*** | [a] Yes<br>[b] No |
| ***In the last 30 days, how frequently have you used insect repellents (include burning mosquito coil and local herbs)?*** | [a] Never<br>[b] Less than once per week<br>[c] 1-3 times per week<br>[d] 4-6 times per week<br>[e] Daily or almost daily |
| ***Have you seen any ticks on an animal or person in the most recent ${season2} season?*** | [a] Yes<br>[b] No |
| ***In the most recent ${season2} season, how many times have you found a tick on your body, either attached or unattached?*** | [a] 0<br>[b] 1-5<br>[c] 6-10 |

| Question | Choices |
| --- | --- |
| | [d] 11-12<br>[e] More than 20 |
| ***In the most recent ${season2} season, have you eaten any engorged, attached, or unattached ticks?*** | [a] Yes<br>[b] No |
| ***Are you aware of any of the youth in your family or neighbors' families eating ticks the most recent ${season2} season?*** | [a] Yes<br>[b] No |
| ***19b. In the most recent ${season2} season, how frequently have you used insect repellents (include burning mosquito coil and local herbs)?*** | [a] Never<br>[b] Less than once per week<br>[c] 1-3 times per week<br>[d] 4-6 times per week<br>[e] Daily or almost daily |
| . | Section Name |
| ***In the last 30 days, how frequently did you engage in any of these activities?*** | |
| ***Livestock production*** | [0] Never<br>[1] Less than once per week<br>[2] 1-3 times per week<br>[3] 4-6 times per week<br>[4] Daily or almost daily |
| ***Crop farming*** | [0] Never<br>[1] Less than once per week<br>[2] 1-3 times per week<br>[3] 4-6 times per week<br>[4] Daily or almost daily |
| ***Hunting*** | [0] Never<br>[1] Less than once per week<br>[2] 1-3 times per week<br>[3] 4-6 times per week<br>[4] Daily or almost daily |
| ***Slaughter or Butchering*** | [0] Never<br>[1] Less than once per week<br>[2] 1-3 times per week<br>[3] 4-6 times per week<br>[4] Daily or almost daily |
| ***Milking*** | [0] Never<br>[1] Less than once per week<br>[2] 1-3 times per week<br>[3] 4-6 times per week<br>[4] Daily or almost daily |
| ***Bleeding livestock*** | [0] Never<br>[1] Less than once per week<br>[2] 1-3 times per week |

| Question | Choices | |
|---|---|---|
| | [3] 4-6 times per week<br>[4] Daily or almost daily | |
| ***Would the trend described above be different in the in the ${season2} season?*** | [a] Yes<br>[b] No | |
| . | | Section Name |
| ***In the most recent ${season2} season, how frequently did you engage in any of these activities?*** | | |
| ***Livestock production*** | [0] Never<br>[1] Less than once per week<br>[2] 1-3 times per week<br>[3] 4-6 times per week<br>[4] Daily or almost daily | |
| ***Crop farming*** | [0] Never<br>[1] Less than once per week<br>[2] 1-3 times per week<br>[3] 4-6 times per week<br>[4] Daily or almost daily | |
| ***Hunting*** | [0] Never<br>[1] Less than once per week<br>[2] 1-3 times per week<br>[3] 4-6 times per week<br>[4] Daily or almost daily | |
| ***Slaughter or Butchering*** | [0] Never<br>[1] Less than once per week<br>[2] 1-3 times per week<br>[3] 4-6 times per week<br>[4] Daily or almost daily | |
| ***Milking*** | [0] Never<br>[1] Less than once per week<br>[2] 1-3 times per week<br>[3] 4-6 times per week<br>[4] Daily or almost daily | |
| ***Bleeding livestock*** | [0] Never<br>[1] Less than once per week<br>[2] 1-3 times per week<br>[3] 4-6 times per week<br>[4] Daily or almost daily | |
| ***Where did the slaughtering and butchering of animals take place? [Multiple Choice]*** | [a] Within or near the household<br>[b] Shared slaughter location within village/town<br>[c] Official slaughterhouse/abattoir<br>[d] Other | |
| ***Explain*** | [Text] | |

| Question | Choices |
| --- | --- |
| ***25. When slaughtering/butchering animals, what types of PPE did you wear? [Multiple Choice]*** | [a] Gloves<br>[b] Goggles<br>[c] Mask<br>[d] Gown/Coat/Apron<br>[e] Boots or shoe covers<br>[f] None |
| ***When slaughtering/butchering animals, what happened to the viscera (blood, organs, skin, sinews, etc.)? [Multiple Choice]*** | [a] Sold<br>[b] Thrown into refuse bin<br>[c] Thrown into the street/left in field or yard<br>[d] Used for household consumption<br>[e] Fed to animals |
| ***When milking animals, what types of PPE did you wear? [Multiple Choice]*** | [a] Gloves<br>[b] Goggles<br>[c] Mask<br>[d] Gown/Coat/Apron<br>[e] Boots or shoe covers<br>[f] None<br>[g] Unsure / don't remember |
| ***When bleeding animals, what types of PPE did you wear? [Multiple Choice]*** | [a] Gloves<br>[b] Goggles<br>[c] Mask<br>[d] Gown/Coat/Apron<br>[e] Boots or shoe covers<br>[f] None<br>[g] Unsure / don't remember |
| . | Section Name |
| ***In the past 30 days, how frequently did you consume meat from the following sources?*** | |
| ***Livestock raised by members of your household*** | [0] Never<br>[1] Less than once per week<br>[2] 1-3 times per week<br>[3] 4-6 times per week<br>[4] Daily or almost daily |
| ***Livestock raised by friends, neighbors or extended family*** | [0] Never<br>[1] Less than once per week<br>[2] 1-3 times per week<br>[3] 4-6 times per week<br>[4] Daily or almost daily |
| ***Local Market / Store / Restaurant*** | [0] Never<br>[1] Less than once per week<br>[2] 1-3 times per week<br>[3] 4-6 times per week<br>[4] Daily or almost daily |
| ***Hunting wild mammals*** | [0] Never<br>[1] Less than once per week<br>[2] 1-3 times per week |

| Question | Choices | |
|---|---|---|
| | [3] 4-6 times per week<br>[4] Daily or almost daily | |
| . | | Section Name |
| ***In the past 30 days, how frequently did you consume meat with the following preparations?*** | | |
| ***Raw*** | [0] Never<br>[1] Less than once per week<br>[2] 1-3 times per week<br>[3] 4-6 times per week<br>[4] Daily or almost daily | |
| ***Cooked*** | [0] Never<br>[1] Less than once per week<br>[2] 1-3 times per week<br>[3] 4-6 times per week<br>[4] Daily or almost daily | |
| ***Cured/dried/salted*** | [0] Never<br>[1] Less than once per week<br>[2] 1-3 times per week<br>[3] 4-6 times per week<br>[4] Daily or almost daily | |
| ***Other*** | [0] Never<br>[1] Less than once per week<br>[2] 1-3 times per week<br>[3] 4-6 times per week<br>[4] Daily or almost daily | |
| ***Explain*** | [Text] | |
| . | | Section Name |
| ***In the past 30 days, how frequently did you consume bird meat from the following sources?*** | | |
| ***Poultry raised by members of your household*** | [0] Never<br>[1] Less than once per week<br>[2] 1-3 times per week<br>[3] 4-6 times per week<br>[4] Daily or almost daily | |
| ***Poultry raised by friends, neighbors or extended family*** | [0] Never<br>[1] Less than once per week<br>[2] 1-3 times per week<br>[3] 4-6 times per week<br>[4] Daily or almost daily | |
| ***Local Market / Store / Restaurant*** | [0] Never<br>[1] Less than once per week<br>[2] 1-3 times per week | |

| Question | Choices | |
|---|---|---|
| | [3] 4-6 times per week<br>[4] Daily or almost daily | |
| ***Hunting wild birds*** | [0] Never<br>[1] Less than once per week<br>[2] 1-3 times per week<br>[3] 4-6 times per week<br>[4] Daily or almost daily | |
| . | | Section Name |
| ***In the past 30 days, how frequently did you consume bird meat with the following preparations?*** | | |
| ***Raw*** | [0] Never<br>[1] Less than once per week<br>[2] 1-3 times per week<br>[3] 4-6 times per week<br>[4] Daily or almost daily | |
| ***Cooked*** | [0] Never<br>[1] Less than once per week<br>[2] 1-3 times per week<br>[3] 4-6 times per week<br>[4] Daily or almost daily | |
| ***Grilled*** | [0] Never<br>[1] Less than once per week<br>[2] 1-3 times per week<br>[3] 4-6 times per week<br>[4] Daily or almost daily | |
| ***Other*** | [0] Never<br>[1] Less than once per week<br>[2] 1-3 times per week<br>[3] 4-6 times per week<br>[4] Daily or almost daily | |
| ***Explain*** | [Text] | |
| . | | Section Name |
| ***In the past 30 days, how frequently did you consume blood products from the following sources?*** | | |
| ***Animals raised by members of your household*** | [0] Never<br>[1] Less than once per week<br>[2] 1-3 times per week<br>[3] 4-6 times per week<br>[4] Daily or almost daily | |
| ***Animals raised by friends, neighbors or extended family*** | [0] Never<br>[1] Less than once per week<br>[2] 1-3 times per week | |

| Question | Choices |
| --- | --- |
| | [3] 4-6 times per week<br>[4] Daily or almost daily |
| ***Local Market / Store / Restaurant*** | [0] Never<br>[1] Less than once per week<br>[2] 1-3 times per week<br>[3] 4-6 times per week<br>[4] Daily or almost daily |
| ***Hunting wild animals*** | [0] Never<br>[1] Less than once per week<br>[2] 1-3 times per week<br>[3] 4-6 times per week<br>[4] Daily or almost daily |
| . | Section Name |
| ***In the past 30 days, how frequently did you consume blood products with the following preparations?*** | |
| ***Raw*** | [0] Never<br>[1] Less than once per week<br>[2] 1-3 times per week<br>[3] 4-6 times per week<br>[4] Daily or almost daily |
| ***Cooked*** | [0] Never<br>[1] Less than once per week<br>[2] 1-3 times per week<br>[3] 4-6 times per week<br>[4] Daily or almost daily |
| ***Other*** | [0] Never<br>[1] Less than once per week<br>[2] 1-3 times per week<br>[3] 4-6 times per week<br>[4] Daily or almost daily |
| ***Explain*** | [Text] |
| . | Section Name |
| ***In the past 30 days, how frequently did you consume milk from the following sources?*** | |
| ***Livestock raised by members of your household*** | [0] Never<br>[1] Less than once per week<br>[2] 1-3 times per week<br>[3] 4-6 times per week<br>[4] Daily or almost daily |
| ***Livestock raised by friends, neighbors or extended family*** | [0] Never<br>[1] Less than once per week<br>[2] 1-3 times per week |

| Question | Choices | |
|---|---|---|
| | [3] 4-6 times per week<br>[4] Daily or almost daily | |
| ***Local Market / Store / Restaurant*** | [0] Never<br>[1] Less than once per week<br>[2] 1-3 times per week<br>[3] 4-6 times per week<br>[4] Daily or almost daily | |
| . | | Section Name |
| ***In the past 30 days, how frequently did you consume milk with the following preparations?*** | | |
| ***Raw, unprocessed*** | [0] Never<br>[1] Less than once per week<br>[2] 1-3 times per week<br>[3] 4-6 times per week<br>[4] Daily or almost daily | |
| ***Boiled or cooked*** | [0] Never<br>[1] Less than once per week<br>[2] 1-3 times per week<br>[3] 4-6 times per week<br>[4] Daily or almost daily | |
| ***Commercially processed*** | [0] Never<br>[1] Less than once per week<br>[2] 1-3 times per week<br>[3] 4-6 times per week<br>[4] Daily or almost daily | |
| ***Fermented or cultured*** | [0] Never<br>[1] Less than once per week<br>[2] 1-3 times per week<br>[3] 4-6 times per week<br>[4] Daily or almost daily | |
| ***Other*** | [0] Never<br>[1] Less than once per week<br>[2] 1-3 times per week<br>[3] 4-6 times per week<br>[4] Daily or almost daily | |
| ***Explain*** | [Text] | |
| ***37. In the past 12 months, how often have you handled live wild animals?*** | [a] Never<br>[b] Less than once per month<br>[c] 1-3 times per month<br>[d] 4-12 times per month<br>[e] 4-6 times per week<br>[f] Daily or multiple times per day | |
| ***38. In the past 12 months, how often have you handled dead wild animals?*** | [a] Never<br>[b] Less than once per month | |

| Question | Choices |
| --- | --- |
| | [c] 1-3 times per month<br>[d] 4-12 times per month<br>[e] 4-6 times per week<br>[f] Daily or multiple times per day |
| ***Explain*** | [Text] |
| ***39. When dead wild animals were handled, what did you do with them? [Multiple Choice]*** | [a] Dispose of or bury carcass<br>[b] Take home for human consumption<br>[c] Take home for animal consumption<br>[d] Use in medicine<br>[e] Other |
| ***40. When dead wild animals were handled, which types of PPE did you wear? [Multiple Choice]*** | [a] Gloves<br>[b] Goggles<br>[c] Mask<br>[d] Gown/Coat/Apron<br>[e] Boots or shoe covers<br>[f] None<br>[g] Unsure / don't remember |
| ***In the past 12 months, which of these species of wild animal did you consume? [Multiple Choice]*** | [a] Warthogs<br>[b] Antelope<br>[c] Bush pigs<br>[d] Rats/Rodents<br>[e] Rabbits<br>[f] Guinea fowl<br>[g] Ostrich<br>[h] None<br>[i] Other |
| ***Explain*** | [Text] |
| . | Section Name |
| ***In the past 12 months, how frequently did you consume wild meat from the following species?*** | |
| ***Warthogs*** | [0] Never<br>[1] Less than once per week<br>[2] 1-3 times per week<br>[3] 4-6 times per week<br>[4] Daily or almost daily |
| ***Antelope*** | [0] Never<br>[1] Less than once per week<br>[2] 1-3 times per week<br>[3] 4-6 times per week<br>[4] Daily or almost daily |
| ***Bush pigs*** | [0] Never<br>[1] Less than once per week<br>[2] 1-3 times per week |

| Question | Choices | |
|---|---|---|
| | [3] 4-6 times per week<br>[4] Daily or almost daily | |
| ***Rats/Rodents*** | [0] Never<br>[1] Less than once per week<br>[2] 1-3 times per week<br>[3] 4-6 times per week<br>[4] Daily or almost daily | |
| ***Rabbits*** | [0] Never<br>[1] Less than once per week<br>[2] 1-3 times per week<br>[3] 4-6 times per week<br>[4] Daily or almost daily | |
| ***Guinea fowl*** | [0] Never<br>[1] Less than once per week<br>[2] 1-3 times per week<br>[3] 4-6 times per week<br>[4] Daily or almost daily | |
| ***Ostrich*** | [0] Never<br>[1] Less than once per week<br>[2] 1-3 times per week<br>[3] 4-6 times per week<br>[4] Daily or almost daily | |
| ***${qn41_explain}*** | [0] Never<br>[1] Less than once per week<br>[2] 1-3 times per week<br>[3] 4-6 times per week<br>[4] Daily or almost daily | |
| ***In the past 12 months, how many times did you have an illness with a fever?*** | [a] 0<br>[b] 1<br>[c] 2<br>[d] 3<br>[e] 4<br>[f] 5<br>[g] 6 or more | |
| . | | Section Name |
| ***In the past 12 months, how many times did you experience an illness with a fever and any of the following additional symptoms?*** | | |
| ***Severe headache*** | [a] 0<br>[b] 1<br>[c] 2<br>[d] 3<br>[e] 4<br>[f] 5<br>[g] 6+<br>[h] Unsure / don’t remember | |

| Question | Choices |
| --- | --- |
| ***Muscle or joint pain*** | [a] 0<br>[b] 1<br>[c] 2<br>[d] 3<br>[e] 4<br>[f] 5<br>[g] 6+<br>[h] Unsure / don't remember |
| ***Vomiting or Diarrhea*** | [a] 0<br>[b] 1<br>[c] 2<br>[d] 3<br>[e] 4<br>[f] 5<br>[g] 6+<br>[h] Unsure / don't remember |
| ***Dizziness or confusion*** | [a] 0<br>[b] 1<br>[c] 2<br>[d] 3<br>[e] 4<br>[f] 5<br>[g] 6+<br>[h] Unsure / don't remember |
| ***Purple or red spots on skin*** | [a] 0<br>[b] 1<br>[c] 2<br>[d] 3<br>[e] 4<br>[f] 5<br>[g] 6+<br>[h] Unsure / don't remember |
| ***Larger bruises*** | [a] 0<br>[b] 1<br>[c] 2<br>[d] 3<br>[e] 4<br>[f] 5<br>[g] 6+<br>[h] Unsure / don't remember |
| ***Nosebleeds*** | [a] 0<br>[b] 1<br>[c] 2<br>[d] 3<br>[e] 4<br>[f] 5<br>[g] 6+<br>[h] Unsure / don't remember |
| ***Bleeding from gums*** | [a] 0<br>[b] 1 |

| Question | Choices | |
|---|---|---|
| | [c] 2<br>[d] 3<br>[e] 4<br>[f] 5<br>[g] 6+<br>[h] Unsure / don't remember | |
| ***Blood in vomit, urine, or stool*** | [a] 0<br>[b] 1<br>[c] 2<br>[d] 3<br>[e] 4<br>[f] 5<br>[g] 6+<br>[h] Unsure / don't remember | |
| ***Other*** | [a] 0<br>[b] 1<br>[c] 2<br>[d] 3<br>[e] 4<br>[f] 5<br>[g] 6+<br>[h] Unsure / don't remember | |
| ***Explain*** | [Text] | |
| . | | Section Name |
| ***Did any of the illnesses that included the symptoms listed above happen after contact with any of the following?*** | | |
| ***Tick bite or contact with blood from a tick*** | [a] Yes<br>[b] No<br>[c] I don't know | |
| ***Raw meat*** | [a] Yes<br>[b] No<br>[c] I don't know | |
| ***Livestock*** | [a] Yes<br>[b] No<br>[c] I don't know | |
| ***Dead wild animal*** | [a] Yes<br>[b] No<br>[c] I don't know | |
| ***In the past 12 months, how many times did members of your household have an illness with a fever?*** | [a] 0<br>[b] 1<br>[c] 2<br>[d] 3<br>[e] 4<br>[f] 5 | |

| Question | Choices | |
|---|---|---|
| | [g] 6 or more<br>[h] Unsure / don't remember | |
| . | | Section Name |
| ***In the past 12 months, how many times did members of your household experience an illness with a fever and any of the following additional symptoms?*** | | |
| ***Severe headache*** | [a] 0<br>[b] 1<br>[c] 2<br>[d] 3<br>[e] 4<br>[f] 5<br>[g] 6+<br>[h] Unsure / don't remember | |
| ***Muscle or joint pain*** | [a] 0<br>[b] 1<br>[c] 2<br>[d] 3<br>[e] 4<br>[f] 5<br>[g] 6+<br>[h] Unsure / don't remember | |
| ***Vomiting or Diarrhea*** | [a] 0<br>[b] 1<br>[c] 2<br>[d] 3<br>[e] 4<br>[f] 5<br>[g] 6+<br>[h] Unsure / don't remember | |
| ***Dizziness or confusion*** | [a] 0<br>[b] 1<br>[c] 2<br>[d] 3<br>[e] 4<br>[f] 5<br>[g] 6+<br>[h] Unsure / don't remember | |
| ***Purple or red spots on skin*** | [a] 0<br>[b] 1<br>[c] 2<br>[d] 3<br>[e] 4<br>[f] 5<br>[g] 6+<br>[h] Unsure / don't remember | |

| Question | Choices |
|---|---|
| ***Larger bruises*** | [a] 0<br>[b] 1<br>[c] 2<br>[d] 3<br>[e] 4<br>[f] 5<br>[g] 6+<br>[h] Unsure / don't remember |
| ***Nosebleeds*** | [a] 0<br>[b] 1<br>[c] 2<br>[d] 3<br>[e] 4<br>[f] 5<br>[g] 6+<br>[h] Unsure / don't remember |
| ***Bleeding from gums*** | [a] 0<br>[b] 1<br>[c] 2<br>[d] 3<br>[e] 4<br>[f] 5<br>[g] 6+<br>[h] Unsure / don't remember |
| ***Blood in vomit, urine, or stool*** | [a] 0<br>[b] 1<br>[c] 2<br>[d] 3<br>[e] 4<br>[f] 5<br>[g] 6+<br>[h] Unsure / don't remember |
| ***Other*** | [a] 0<br>[b] 1<br>[c] 2<br>[d] 3<br>[e] 4<br>[f] 5<br>[g] 6+<br>[h] Unsure / don't remember |
| ***Explain*** | [Text] |
| . | Section Name |
| ***Did any of the illnesses that included the symptoms listed above happen after contact with any of the following?*** | |

| Question | Choices |
| --- | --- |
| *Tick bite or contact with blood from a tick* | [a] Yes<br>[b] No<br>[c] I don't know |
| *Raw meat* | [a] Yes<br>[b] No<br>[c] I don't know |
| *Livestock* | [a] Yes<br>[b] No<br>[c] I don't know |
| *Wild animal* | [a] Yes<br>[b] No<br>[c] I don't know |
| . | Section Name |
| *In the past 12 months, how often have you physically interacted with any of the following animals? Include interactions with animals you own as well as those you do not own.* | |
| *Cattle/Buffalo* | [0] Never<br>[1] Less than once per week<br>[2] 1-3 times per week<br>[3] 4-6 times per week<br>[4] Daily or almost daily |
| *Camel* | [0] Never<br>[1] Less than once per week<br>[2] 1-3 times per week<br>[3] 4-6 times per week<br>[4] Daily or almost daily |
| *Horse* | [0] Never<br>[1] Less than once per week<br>[2] 1-3 times per week<br>[3] 4-6 times per week<br>[4] Daily or almost daily |
| *Donkey/mule* | [0] Never<br>[1] Less than once per week<br>[2] 1-3 times per week<br>[3] 4-6 times per week<br>[4] Daily or almost daily |
| *Goat/sheep* | [0] Never<br>[1] Less than once per week<br>[2] 1-3 times per week<br>[3] 4-6 times per week<br>[4] Daily or almost daily |
| *Pig/swine* | [0] Never<br>[1] Less than once per week |

| Question | Choices |
|---|---|
| | [2] 1-3 times per week<br>[3] 4-6 times per week<br>[4] Daily or almost daily |
| ***Chicken*** | [0] Never<br>[1] Less than once per week<br>[2] 1-3 times per week<br>[3] 4-6 times per week<br>[4] Daily or almost daily |
| ***Turkey/duck/goose*** | [0] Never<br>[1] Less than once per week<br>[2] 1-3 times per week<br>[3] 4-6 times per week<br>[4] Daily or almost daily |
| ***Dog*** | [0] Never<br>[1] Less than once per week<br>[2] 1-3 times per week<br>[3] 4-6 times per week<br>[4] Daily or almost daily |
| ***Cat*** | [0] Never<br>[1] Less than once per week<br>[2] 1-3 times per week<br>[3] 4-6 times per week<br>[4] Daily or almost daily |
| . | Section Name |
| ***In the past 12 months, how many of each of the following types of animals have you owned?*** | |
| ***Cattle/Buffalo*** | [Integer] |
| ***Camel*** | [Integer] |
| ***Horse*** | [Integer] |
| ***Donkey/mule*** | [Integer] |
| ***Goat/sheep*** | [Integer] |
| ***Pig/swine*** | [Integer] |
| ***Chicken*** | [Integer] |
| ***Turkey/duck/goose*** | [Integer] |
| ***Dog*** | [Integer] |
| ***Cat*** | [Integer] |

| Question | Choices | |
|---|---|---|
| . | | Section Name |
| ***In the past 12 months, how often did you provide treatment to your animals for ticks such as spray, dip, or injection?*** | | |
| ***Cattle/Buffalo*** | [0] Never<br>[1] Less than Monthly<br>[2] Monthly<br>[3] Weekly<br>[4] Daily<br>[5] As Needed | |
| ***Camel*** | [0] Never<br>[1] Less than Monthly<br>[2] Monthly<br>[3] Weekly<br>[4] Daily<br>[5] As Needed | |
| ***Horse*** | [0] Never<br>[1] Less than Monthly<br>[2] Monthly<br>[3] Weekly<br>[4] Daily<br>[5] As Needed | |
| ***Donkey/mule*** | [0] Never<br>[1] Less than Monthly<br>[2] Monthly<br>[3] Weekly<br>[4] Daily<br>[5] As Needed | |
| ***Goat/sheep*** | [0] Never<br>[1] Less than Monthly<br>[2] Monthly<br>[3] Weekly<br>[4] Daily<br>[5] As Needed | |
| ***Pig/swine*** | [0] Never<br>[1] Less than Monthly<br>[2] Monthly<br>[3] Weekly<br>[4] Daily<br>[5] As Needed | |
| ***Chicken*** | [0] Never<br>[1] Less than Monthly<br>[2] Monthly<br>[3] Weekly<br>[4] Daily<br>[5] As Needed | |

| Question | Choices |
| --- | --- |
| ***Turkey/duck/goose*** | [0] Never<br>[1] Less than Monthly<br>[2] Monthly<br>[3] Weekly<br>[4] Daily<br>[5] As Needed |
| ***Dog*** | [0] Never<br>[1] Less than Monthly<br>[2] Monthly<br>[3] Weekly<br>[4] Daily<br>[5] As Needed |
| ***Cat*** | [0] Never<br>[1] Less than Monthly<br>[2] Monthly<br>[3] Weekly<br>[4] Daily<br>[5] As Needed |
| . | Section Name |
| ***In the past 12 months, which methods did you utilize to treat your animals for ticks?*** | |
| ***Choice [Multiple Choice]*** | [a] Spray<br>[b] Dip<br>[c] Injection<br>[d] Other |
| ***Cattle/Buffalo [Multiple Choice]*** | [a] Spray<br>[b] Dip<br>[c] Injection<br>[d] Other |
| ***Camel [Multiple Choice]*** | [a] Spray<br>[b] Dip<br>[c] Injection<br>[d] Other |
| ***Horse [Multiple Choice]*** | [a] Spray<br>[b] Dip<br>[c] Injection<br>[d] Other |
| ***Donkey/mule [Multiple Choice]*** | [a] Spray<br>[b] Dip<br>[c] Injection<br>[d] Other |
| ***Goat/sheep [Multiple Choice]*** | [a] Spray<br>[b] Dip |

| Question | Choices | |
|---|---|---|
| | [c] Injection<br>[d] Other | |
| *Pig/swine [Multiple Choice]* | [a] Spray<br>[b] Dip<br>[c] Injection<br>[d] Other | |
| *Chicken [Multiple Choice]* | [a] Spray<br>[b] Dip<br>[c] Injection<br>[d] Other | |
| *Turkey/duck/goose [Multiple Choice]* | [a] Spray<br>[b] Dip<br>[c] Injection<br>[d] Other | |
| *Dog [Multiple Choice]* | [a] Spray<br>[b] Dip<br>[c] Injection<br>[d] Other | |
| *Cat [Multiple Choice]* | [a] Spray<br>[b] Dip<br>[c] Injection<br>[d] Other | |
| *Explain* | [Text] | |
| . | | Section Name |
| *In the past 12 months, how often did you manually remove ticks from your animals?* | | |
| *Cattle/Buffalo* | [0] Never<br>[1] Less than Monthly<br>[2] Monthly<br>[3] Weekly<br>[4] Daily<br>[5] As Needed | |
| *Camel* | [0] Never<br>[1] Less than Monthly<br>[2] Monthly<br>[3] Weekly<br>[4] Daily<br>[5] As Needed | |
| *Horse* | [0] Never<br>[1] Less than Monthly<br>[2] Monthly<br>[3] Weekly<br>[4] Daily<br>[5] As Needed | |

| Question | Choices |
| --- | --- |
| ***Donkey/mule*** | [0] Never<br>[1] Less than Monthly<br>[2] Monthly<br>[3] Weekly<br>[4] Daily<br>[5] As Needed |
| ***Goat/sheep*** | [0] Never<br>[1] Less than Monthly<br>[2] Monthly<br>[3] Weekly<br>[4] Daily<br>[5] As Needed |
| ***Pig/swine*** | [0] Never<br>[1] Less than Monthly<br>[2] Monthly<br>[3] Weekly<br>[4] Daily<br>[5] As Needed |
| ***Chicken*** | [0] Never<br>[1] Less than Monthly<br>[2] Monthly<br>[3] Weekly<br>[4] Daily<br>[5] As Needed |
| ***Turkey/duck/goose*** | [0] Never<br>[1] Less than Monthly<br>[2] Monthly<br>[3] Weekly<br>[4] Daily<br>[5] As Needed |
| ***Dog*** | [0] Never<br>[1] Less than Monthly<br>[2] Monthly<br>[3] Weekly<br>[4] Daily<br>[5] As Needed |
| ***Cat*** | [0] Never<br>[1] Less than Monthly<br>[2] Monthly<br>[3] Weekly<br>[4] Daily<br>[5] As Needed |
| ***Do you wear gloves when you remove ticks from your animals?*** | [a] Always<br>[b] Sometimes<br>[c] Never |
| . | Section Name |

| Question | Choices |
| --- | --- |
| ***55. Please indicate which of the following are true about one or more of the animals you have owned in the past 12 months*** | |
| ***Choice [Multiple Choice]*** | [a] Became Ill<br>[b] Died from illness<br>[c] None |
| ***Cattle/Buffalo [Multiple Choice]*** | [a] Became Ill<br>[b] Died from illness<br>[c] None |
| ***Camel [Multiple Choice]*** | [a] Became Ill<br>[b] Died from illness<br>[c] None |
| ***Horse [Multiple Choice]*** | [a] Became Ill<br>[b] Died from illness<br>[c] None |
| ***Donkey/mule [Multiple Choice]*** | [a] Became Ill<br>[b] Died from illness<br>[c] None |
| ***Goat/sheep [Multiple Choice]*** | [a] Became Ill<br>[b] Died from illness<br>[c] None |
| ***Pig/swine [Multiple Choice]*** | [a] Became Ill<br>[b] Died from illness<br>[c] None |
| ***Chicken [Multiple Choice]*** | [a] Became Ill<br>[b] Died from illness<br>[c] None |
| ***Turkey/duck/goose [Multiple Choice]*** | [a] Became Ill<br>[b] Died from illness<br>[c] None |
| ***Dog [Multiple Choice]*** | [a] Became Ill<br>[b] Died from illness<br>[c] None |
| ***Cat [Multiple Choice]*** | [a] Became Ill<br>[b] Died from illness<br>[c] None |
| ***In the past 12 months, what did you do when an animal became ill? Select all that apply. [Multiple Choice]*** | [a] Slaughter the animal, butcher and consume the meat<br>[b] Slaughter the animal and sell the meat<br>[c] Kill the animal and dispose of the carcass<br>[d] Sell the live animal<br>[e] Get veterinary care<br>[f] Report to authorities<br>[g] Other |

| Question | Choices | |
|---|---|---|
| ***Explain*** | [Text] | |
| . | | Section Name |
| ***Please indicate the number of animals that died from illness in the past 12 months.*** | | |
| ***Cattle/Buffalo*** | [Integer] | |
| ***Camel*** | [Integer] | |
| ***Horse*** | [Integer] | |
| ***Donkey/mule*** | [Integer] | |
| ***Goat/sheep*** | [Integer] | |
| ***Pig/swine*** | [Integer] | |
| ***Chicken*** | [Integer] | |
| ***Turkey/duck/goose*** | [Integer] | |
| ***Dog*** | [Integer] | |
| ***Cat*** | [Integer] | |
| ***In the past 12 months, what did you do when an animal died from a disease? Select all that apply. [Multiple Choice]*** | [a] Bury the carcass<br>[b] Dump the carcass<br>[c] Feed carcass to dogs<br>[d] Butcher and consume the meat uncooked<br>[e] Butcher and consume the meat cooked<br>[f] Other | |
| ***Explain*** | [Text] | |
| ***Please select the answer that best describes how you have grazed your livestock in the past 12 months?*** | [a] In a herd with only animals from my household<br>[b] In a herd with animals from multiple households<br>[c] Both | |
| ***Please select the answer that best describes how you have grazed your livestock in the past 12 months.*** | [a] In a location with frequent presence of wildlife<br>[b] In a location with infrequent presence of wildlife<br>[c] Both | |
| ***In the past 12 months, what is the greatest distance have you travelled while grazing your livestock?*** | [a] Less than 10 km – Movements within a localized area (eg, between nearby grazing areas and water sources)<br>[b] 10–50 km – Short-distance seasonal movement within the same district<br>[c] 51–100 km – Moderate migration across districts but within the same region<br>[d] 101–300 km – Long-distance movement across regions in search of pasture and water | |

| Question | Choices |
| --- | --- |
| | [e] More than 300 km (within Tanzania) – Extensive migration across multiple regions but staying within the country<br>[f] More than 300 km (including crossing into neighboring countries) – Migration that includes movement into Kenya, Uganda, Rwanda, Burundi, Zambia, or Mozambique |
| ***In the past 12 months, from where have you obtained new livestock? [Multiple Choice]*** | [a] From births within my herd/flock<br>[b] From friends, neighbors, or relatives<br>[c] From markets<br>[d] Not applicable |
| ***Did you treat these new animals for ticks before mixing them with your herd/flock? [Multiple Choice]*** | [a] Yes, all<br>[b] Yes, some<br>[c] No |
| ***Data submission without consent is not permitted.*** | |
